# Tracheostomy in Critically Ill COVID-19 Patients on Extracorporeal Membrane Oxygenation: A Single-Center Experience

**DOI:** 10.1177/00034894231166648

**Published:** 2023-04-09

**Authors:** Phillip Staibano, Shahzaib Khattak, Faizan Amin, Paul T. Engels, Doron D. Sommer

**Affiliations:** 1 Department of Surgery, Division of Otolaryngology–Head and Neck Surgery, McMaster University, Hamilton, ON, Canada; 2 Department of Medicine, Division of Cardiology, McMaster University, Hamilton, ON, Canada; 3 Department of Medicine, Division of Critical Care, McMaster University, Hamilton, ON, Canada; 4 Department of Surgery, Division of General Surgery, McMaster University, Hamilton, ON, Canada

**Keywords:** tracheostomy, COVID-19, ECMO, patient outcomes

## Abstract

**Objectives:**

Novel coronavirus-19 (COVID-19) has led to over 6 million fatalities globally. An estimated 75% of COVID-19 patients who require critical care admission develop acute respiratory distress syndrome (ARDS) needing invasive mechanical ventilation (IMV) and/or extracorporeal membrane oxygenation (ECMO). Due to prolonged ventilation requirements, these patients often also require tracheostomy. We performed a review of clinical outcomes in COVID-19 patients on ECMO at a high-volume tertiary care center in Hamilton, Ontario, Canada.

**Methodology:**

We performed a retrospective case series, including 24 adult patients diagnosed with COVID-19 who required IMV, veno-venous (ECMO), and tracheostomy. All patients were included from April to December 2021. We extracted demographic and clinical variables pertaining to the tracheostomy procedure and ECMO therapy. We performed descriptive statistical analyses. This study was approved by the Hamilton Integrated Research Ethics Board (14217-C).

**Results:**

We included 24 consecutive patients with COVID-19 who required tracheostomy while undergoing ECMO therapy. The mean age was 49.4 years [standard deviation (SD): 7.33], the majority of patients were male (75%), with mean body mass index of 32 (SD: 8.81). Overall mortality rate was 33.3%. Percutaneous tracheostomy was performed most frequently (83.3%) and, similar to open tracheostomy, was associated with a low rate of perioperative bleeding complications. Within surviving patients, the mean time to IMV weaning and decannulation was 60.2 (SD: 24.6) and 49.4 days (SD: 21.8), respectively.

**Conclusion:**

Percutaneous tracheostomy appears to be safe in COVID-19 patients on ECMO and holding anticoagulation 24 hours prior to and after tracheostomy may limit bleeding events in these patients.

## Introduction

As of February 19, 2023, over 674 million patients have been diagnosed with the novel coronavirus 2019 (COVID-19) and over 6.8 million individuals have died around the world.^1^ The vast majority of patients infected by the novel coronavirus 2019 (SARS-CoV-2) develop mild upper respiratory tract symptoms. However, 20% of patients who are hospitalized with COVID-19, typically those with known risk factors such as age over 65 years, obesity, or additional medical comorbidities, require critical care admission.^2,3^ An estimated 75% of critically ill COVID-19 patients develop acute respiratory distress syndrome (ARDS), which is a life-threatening complication that requires invasive mechanical ventilation (IMV) and often tracheostomy.^4-6^ Early tracheostomy in COVID-19 patients may be associated with shortened critical care length of stay.^6,7^ The mortality rate of COVID-19 ARDS remains high, however, with global estimates ranging from 13% to 73%.^8^ Prior to the COVID-19 pandemic, extracorporeal membrane oxygenation (ECMO) had been used in critically ill ARDS patients to assist with pulmonary recovery by oxygenating venous blood via an artificial membrane oxygenator.^9,10^ The Extracorporeal Life Support Organization (ELSO), along with other critical care organizations, have recommended ECMO for COVID-19 patients based on existing guidelines that have used veno-venous ECMO for other causes of ARDS.^11^ Prognosis of COVID-19 patients that receive ECMOdependent on several factors, including patient selection, disease severity, and adjunctive treatments.^12^ In Canada and other global centers, access to ECMO resources has been a challenge during peaks of the COVID-19 pandemic.^13^ Herein, we aimed to describe the clinical features and outcomes of critically ill COVID-19 patients who underwent ECMO cannulation and tracheostomy at a single high-volume, tertiary care institution in Canada.

## Material and Methods

### Study design

We performed a retrospective case series analysis of consecutive COVID-19 patients admitted to the intensive care unit (ICU) at the Hamilton General Hospital (Hamilton, Ontario, Canada) from April to December 2021. We performed this study during the height of the third COVID-19 wave in Ontario, Canada, where infections were largely driven initially by the alpha variant and later by the delta variant and did overwhelm healthcare resources, often requiring inter-provincial transfer of patients to high-volume centers; hence, the third wave represented a critical time wherein our highest quota of ICU beds and ECMO machines were devoted to COVID-19 patients. We performed this study during the height of the third COVID-19 wave, which, was associated with a higher mortality rate in patients treated with ECMO as compared to the first wave.^14,15^ This was conducted in accordance with the Preferred Reporting Of Case Series in Surgery (PROCESS) guidelines.^16^ This study was approved by the Hamilton Integrated Research Ethics Board (14217-C).

### Patient eligibility

We reviewed the medical records of COVID-19 patients admitted to the ICU at the Hamilton General Hospital between April and December 2021. The Hamilton General Hospital is a high-volume academic institution in a metropolitan center with a catchment area of approximately 3 million people. We included adult patients (ie, ≥18 years old) who were initially endotracheally intubated for COVID-19 ARDS and required ECMO cannulation followed by tracheostomy during their ICU admission. All diagnoses of ARDS and clinical decisions to undergo IMV were made in conjunction with accepted diagnostic criteria.^17^ The decision for ECMO cannulation was made by the team of critical care physicians and cardiac surgeons on a patient-to-patient basis and in accordance with ELSO guidelines for COVID-19 patients.^9^ Our general criteria for initiating ECMO included patients with hypoxemic respiratory failure and a PaO_2_/FiO_2_ <80 mmHg despite optimal ventilation settings, in addition to a lack of pre-existing conditions incompatible with recovery. For included patients, ECMO cannulation was performed using standard sterile technique and cannulation was typically performed within 10 days of intubation. All patients on ECMO were anticoagulated with intravenous unfractionated heparin using a low-dose protocol guided by anti-Xa levels. The decision to undergo tracheostomy was considered on a patient-to-patient basis by the attending critical care physician and the consulting surgical service and performed in accordance with clinical guidelines.^18^ The clinical indication for undergoing tracheostomy included facilitation of prolonged ventilation weaning and endotracheal intubation for at least 10 to 14 days. We proceeded with tracheostomy in patients who had an PTT<35 seconds, INR/PT <1.5, and platelets >50 × 10^9^/L.^19,20^ All patients underwent open or percutaneous tracheostomy (Blue Rhino, Cook Medical, Bloomington, Indiana, USA) depending on their clinical stability and anatomical landmarking. Percutaneous tracheostomies were performed in the ICU or in the operating suite using sterile technique. All surgeons used personal protective equipment (PPE) appropriate for COVID-19 aerosol-generating medical procedures. All tracheostomies were performed during ECMO therapy and all anticoagulation was stopped in advance of the procedure and held following tracheostomy. Initially, anticoagulation was held at least 4 hours prior to tracheostomy and restarted 4 hours following tracheostomy. In later cases, anticoagulation was held approximately 24 hours prior to surgery and restarted 24 hours after surgery. Laboratory testing for COVID-19 was performed via polymerase chain reaction (PCR) analysis of nasopharyngeal and/or endotracheal tube aspirate and the following variants of concern (VOC) were tested: COVID-19 S, N50Y1 S, E484K S, and B1.617.2. A patient was classified as COVID-19 negative if he/she had at least 1 negative COVID-19 PCR result. We deemed a patient vaccinated against COVID-19 if he/she had at least 2 doses of any COVID-19 vaccine approved for use by Health Canada greater than 2 weeks after COVID-19 diagnosis.

### Data collection and statistical analysis

We reviewed the electronic medical records for eligible patients from April to December 2021. All patients were analyzed from the time of COVID-19 diagnosis and through their duration of ECMO therapy until hospital discharge or death. All medical information was collected using a formal extraction form and was performed by 2 independent reviewers (PS, SK) with verification by senior authors (FA, DDS). We collected patient data including age, gender, and body mass index (BMI), in addition to details surrounding their COVID-19 diagnosis and vaccination status. We also collected data pertaining to the ICU course, in addition to tracheostomy, ECMO details, and relevant biochemical and hematologic bloodwork values (ie, aPTT, PT/INR, anti-Xa, serum fibrinogen, and complete blood count). All laboratory values were collected for the entirety of ECMO duration. The biochemical tests, with the exception of the functional platelet testing, were extracted twice per day for each patient. We performed a descriptive analysis of all included patients, including measures of central tendency and standard deviation (SD), where applicable. For aPTT values >150, we used a maximum value of 150 and for INR <0.1, we used a minimum value of 0.1 for all calculations. All statistical analyses were performed with Microsoft Excel (Redmond, Washington, USA).

## Results

### Demographic and COVID-19-related statistics

All 24 patients were recruited consecutively from the ICU at the Hamilton General Hospital in Hamilton, Ontario, Canada. All patients were admitted to the ICU from April to December 2021 (Table 1). The mean age of all included patients at the time of ECMO cannulation was 49.4 years (SD: 7.33; range: 34-63) and the majority of patients were male (n = 18, 75%). The mean BMI across all patients with recorded values (n = 19) was 32 (SD: 8.81; range: 20.1-54.8). There were 8 patients (33.3%) without any documented medical comorbidities, while remaining patients had multiple medical comorbidities (n = 16, 66.7%). We found that 5 patients were either active or former smokers with a 10 to 30 pack-year history. There was no additional relevant drug or alcohol-use history nor were there any notable drug allergies. Five patients (20.8%) diagnosed with COVID-19 in April 2021 did not test positive for any VOCs while 4 patients (16.7%) had more than 1 VOC (ie, COVID-19 S, N501Y S, and/or B.1.1.7). All patients diagnosed with COVID-19 from July to October 2021 (n = 12, 50%) tested positive for the Delta (B1.617.2) variant. Notably, none of these patients received 2 doses of any COVID-19 vaccine prior to diagnosis. All patients received a single dose of tocilizumab at the time of COVID-19 hospitalization.

**Table 1.** Patient Characteristics.

Characteristic^a^	Value
Age, years	49.4 ± 7.33
Gender	
Male	18 (75)
Female	6 (25)
Body mass index (BMI, kg/m^2^)	32 ± 8.81
Medical comorbidities	
None	8 (33.3)
Hypertension	6 (25)
Dyslipidemia	4 (16.7)
Diabetes mellitus (type 2)	3 (12.5)
Social history	
None	14 (58.3)
Active or former smoking history	5 (20.8)
Not reported	5 (20.8)
COVID-19 diagnosed via NP swab PCR testing	24 (100)
COVID-19 variants of concern (VOC)	29
B1.617.2	12 (41.4)
COVID-19 S	5 (17.2)
N501Y S	5 (17.2)
None	5 (17.2)
B.1.1.7	1 (3.45)
Not reported	1 (3.45)
At least 2 doses of COVID-19 vaccine	0 (0)

Abbreviations: BMI, body mass index (kg/m^2^), NP, nasopharyngeal, PCR, polymerase chain reaction, VOC, variants of concerns.

a Continuous data are presented as the median (range) or mean ± SD; categoric data are presented as number (%).

### Tracheostomy outcomes

All 24 patients were intubated for hypoxic respiratory failure secondary to COVID-19 ARDS at the time of critical care admission. This intubation was either performed on day of admission to our ICU or at a peripheral hospital ICU prior to patient transfer (Table 2). Percutaneous tracheostomy was performed most frequently (n = 20, 83.3%). The indications for open tracheostomy were high BMI and/or difficult neck anatomical landmarks (n = 2) and medical instability associated with active bleeding events (n = 3). The Division of Otolaryngology–head and neck surgery performed all open and the majority of percutaneous tracheostomies. All tracheostomies were confirmed via peri/intra-procedure bronchoscopy. There were 2 documented perioperative bleeding events: 2 during a percutaneous tracheostomy that was controlled with local measuress and 1 in the day following open tracheostomy that was controlled with bedside electrocautery. One patient had severe erosion of his tracheostomy stoma, in addition to localized pseudomonal infection that required long-term surgical packing and local antibiotic therapy. The mean time from intubation to tracheostomy was 25.8 days (SD: 11.3, range: 13-65 days). The mean time from ECMO cannulation to tracheostomy was 19.7 days (SD: 11.3, range: 9-64 days). Within the surviving cohort, the mean time from intubation to IMV weaning was 60.2 days (SD: 24.6, range: 38-96) and the mean time from tracheostomy to decannulation was 49.4 days (SD: 21.8, range: 18-90). No staff member reported COVID-19 transmission associated with performing tracheostomy in this cohort.

**Table 2.** Tracheostomy Characteristics.

Characteristic^a^	Value
Type of tracheostomy	24 (100)
Percutaneous	20 (83.3)
Open	4 (16.7)
Setting of tracheostomy	
Percutaneous^*^	
ICU (bedside)	18 (94.7)
Operating suite	1 (5.26)
Open	
Operating suite	4 (100)
Surgical service	
Otolaryngology–head and neck surgery	20 (83.3)
Cardiac surgery	2 (8.33)
General surgery	1 (4.17)
Not reported	1 (4.17)
Perioperative complications	
Percutaneous	
Bleeding	2 (10)
Open	
Bleeding	1 (25)
Stoma erosion	1 (25)
Time to tracheostomy from ECMO cannulation (days) (n = 24)	19.7 ± 11.3
Time to tracheostomy from IMV (days) (n = 23)	25.8 ± 11.3
Percutaneous (n = 19)	24.9 ± 7.66
Open (n = 4)	29.5 ± 20.7
Time to ventilation wean (days) (n = 9)	60.2 ± 24.6
Percutaneous (n = 6)	61 ± 23.3
Open (n = 3)	58.7 ± 32.3
Time to decannulation (days) (n = 13)	49.4 ± 21.8
Percutaneous (n = 10)	51 ± 23.4
Open (n = 3)	44 ± 17.8

a Continuous data are presented as the median (range) or mean ± SD; categoric data are presented as number (%).

* The setting of percutaneous tracheostomy was not reported in 1 patient.

### Critical care outcomes

In this cohort, 16 patients (66.7%) survived, were successfully decannulated from ECMO, and discharged from hospital. The mean ICU length of stay for all surviving patients was 76.9 days (SD: 36.9, range: 22-144 days), while the mean time to hospital discharge from ICU admission was 118.7 days (SD: 49.4, range: 53-223 days) (Table 3). The most common ICU complication was ventilator-associated pneumonia (n = 17, 70.8%), which was commonly caused by multi-drug resistant *Pseudomonas aeruginosa* (20.8%), but also resulted from *Klebsiella aerogenes* and/or *Escherichia coli*. Seven patients (29.2%) experienced viremia caused by cytomegalovirus and/or herpes simplex virus. Ten patients (41.7%) were treated for bacteremia and there was 1 case of fungal septicemia. Three surviving patients were discharged home on home oxygen therapy.

**Table 3.** Critical Care Characteristics.

Characteristic^a^	Value
No. of patients who died	8 (33.3)
Died during ECMO therapy	7 (87.5)
Died following ECMO therapy	1 (12.5)
Cause of death	
Multi-drug resistant/recurrent sepsis	6 (75)
Subarachnoid hemorrhage	1 (12.5)
Pulmonary embolism	1 (12.5)
ICU length of stay, days (n = 16)	69.1 ± 30.9
Time to hospital discharge, days (n = 16)	118.7 ± 49.4
ICU complications	
Ventilator-associated pneumonia	17 (70.8)
MSSA and/or MRSA bacteremia	10 (41.7)
CMV and/or HSV-1 viremia	7 (29.2)
Acute Kidney injury requiring renal replacement therapy	6 (25)
Duration of ECMO therapy, days (n = 16)	38.9 ± 15.2
Time from IMV to ECMO cannulation, days (n = 23)	5.65 ± 3.58
Bleeding complications	11 (45.8)
Hemothorax	3 (27.3)
Tracheostomy site	3 (27.3)
Epistaxis	2 (18.2)
Subarachnoid hemorrhage	2 (18.2)
Gastrointestinal bleeding	1 (9.1)
Thrombotic complications	13 (54.2)
Deep vein thrombosis	5 (41.7)
Pulmonary embolism	4 (33.3)
Heparin-induced thrombocytopenia	1 (8.33)
Thrombotic gangrene	1 (8.33)
Retroperitoneal hematoma	1 (8.33)
Right ventricle thrombus	1 (8.33)
ECMO laboratory values	
aPTT, s	52.7 ± 12.1
Platelets, 10^9^/L	141 ± 39.8
Anti-Xa, U/mL	0.42 ± 0.148
INR	1.21 ± 0.119
Fibrinogen, g/L	3.44 ± 0.904

Abbreviations: CMV, cytomegalovirus; HSV, herpes simplex virus; IMV, invasive mechanical ventilation; MSSA, Methicillin-sensitive *Staphylococcus aureus*; MRSA, Methicillin-resistant *Staphylococcus aureus*.

a Continuous data are presented as the median (range) or mean ± SD; categoric data are presented as number (%).

For all patients, the mean time from intubation to ECMO cannulation was 5.65 days (SD: 15.2, range: 1-16 days). The mean time of ECMO therapy for surviving patients was 38.9 days (SD: 15.2, range: 16-82 days). We found that there were 11 bleeding events during the critical care course. In addition, there were 13 thrombotic complications (primarily venous) that were treated with therapeutic anticoagulation. Four deep vein thrombotic events occurred in the femoral and/or internal jugular veins, which may have been secondary to ECMO cannulation. One patient developed heparin-induced thrombocytopenia during ECMO therapy and was treated with fondaparinux. Furthermore, we found that during ECMO therapy, the mean laboratory ranges were the following, aPTT: 35.2 to 70.6, platelets: 90.5 to 272, anti-Xa: 0.23 to 0.95, INR: 1.04 to 1.57, and fibrinogen: 1.94 to 5.49. Overall, 8 patients died (33.3%) and of these patients, 7 died prior to ECMO decannulation and 1 patient died following ECMO decannulation and transfer back to the ICU at a peripheral hospital.

## Discussion

The COVID-19 pandemic has led to a devastating healthcare and economic burden around the world.^21^ In a study of >840 000 hospitalized COVID-19 patients, <1% require ECMO and/or tracheostomy with ECMO being performed in younger, male patients with few medical comorbidities and tracheostomy not having a demographic bias.^22^ The estimated mortality rate in COVID-19 patients admitted to critical care can be upwards of 49%.^23^ In COVID-19 patients on ECMO, the mortality rate has varied from the start of pandemic, but is estimated to be 50% to 80% in high-volume centers.^24^ Here, the patient cohort was relatively young with these patients having an obese BMI and several chronic medical comorbidities. Additionally, our mortality rate was lower at 33.3%, which may be due to earlier initiation of ECMO and/or improved patient management and resource allocation during the third wave of the pandemic. These findings may also reflect the improvement of clinical outcomes of COVID-19 patients managed via ECMO since the start of the pandemic.^15,25^ Other studies have shown that relatively healthy and young COVID-19 patients can comprise up to 75% of those requiring ICU admission.^26^ All included patients were not fully vaccinated with at least 2 doses of the COVID-19 vaccine at the time of COVID-19 diagnosis, which may also be due to the public vaccination campaign in Ontario only beginning in January 2021. Marginalized communities in Ontario are more likely to be severely affected by COVID-19 and are less likely to be fully vaccinated against COVID-19.^27^ By August 2021, 72% of Canadians had a single COVID-19 vaccine dose while 61% had received both vaccine doses.^28^ COVID-19 vaccination protects against poor disease outcomes, hospitalization, the risk of requiring critical care admission.^29^ We also found that approximately half of included COVID-19 patients had the SARS CoV-2 delta variant, which is associated with a higher risk of hospitalization and death compared to the SARS CoV-2 alpha variant.^30^ Similar to other studies, we found that a number of COVID-19 patients within this cohort experienced secondary bacterial and/or viral infections, possibly secondary to pathologic and/or iatrogenic immunosuppression.^31,32^ The medical comorbidities, in addition to the prothrombotic state in many COVID-19 patients and the aforementioned high rate of secondary infections may have impacted the timing of ECMO initiation, tracheostomy, and the overall prognosis of patients included in this cohort.

The duration of ECMO therapy for our COVID-19 patient population was similar to previously published estimates.^33^ Our time to ECMO cannulation from intubation was similar; however, this metric has been shown to have no impact on COVID-19 survival.^34^ Despite chemical thromboprophylaxis, critically ill COVID-19 patients have a thrombotic complication rate of 31% to 34%.^35,36^ Moreover, cannula-associated thrombotic events have an incidence as high as 71% during ECMO therapy.^37^ Our cohort demonstrated a slightly elevated thrombosis rate compared to published estimates, but our rates included arterial, cardiac, and venous thrombotic events. Bleeding rates in ECMO are also reported to be as high as 29%, and the slightly higher rate in this study may secondary to relatively high rates of therapeutic anticoagulation within the mileu of the prothrombotic state of COVID-19.^38^ Thus far, COVID-19 patients on ECMO have demonstrated a elevated risk of bleeding events, in particular intracranial hemorrhage (ie, 5-10.5%), and a heightened risk of circuit thrombi, pulmonary embolism, and HIT with thrombotic complications.^39^ For the first 11 patients within our cohort, we held unfractionated heparin 4 hours prior to tracheostomy and restarted anticoagulation 4 hours following the procedure, which is in accordance with previous recommendations.^40^ Three of these patients, however, experienced peristomal bleeding and so, we extended our anticoagulation holding parameters to a minimum 24 hours pre- and post-tracheostomy, which led to fewer bleeding events in this cohort. In studies of non-COVID-19 patients on ECMO, low-dose heparin strategies and heparin holding parameters 4 hours pre- and post-tracheostomy are associated with a low rate of severe bleeding events, but, there is no consensus on anticoagulation holding parameters in COVID-19 patients.^41^ Studies of tracheostomy in COVID-19 patients on ECMO, thus far, have not identified optimal algorithms for anticoagulation. Depending on the patient-specific thrombotic risk and the heparin protocol used during ECMO, we recommend holding heparin for 24 hours prior to tracheostomy and restarting anticoagulation >24 hours after tracheostomy.

In non-COVID-19 ARDS, tracheostomy performed after ECMO cannulation was associated with an increased risk of major bleeding, but not with earlier sedation weaning.^42^ Furthermore, percutaneous tracheostomy in ECMO patients is associated with fewer bleeding events and a similar complication rate to open tracheostomy.^43^ In this cohort, percutaneous tracheostomy was performed more often than open, and there was no qualitative difference in bleeding or complication rates. Here, tracheostomy was performed an average of 19.7 days following ECMO cannulation, with the decision to undergo tracheostomy largely dependent whether the patient had a high likelihood of ECMO decannulation in keeping with national and institutional guidelines.^45^ A multi-center study of 2259 COVID-19 patients identified that tracheostomy performed within 10 days of ECMO cannulation was associated with shortened ECMO duration, but mortality and mobilization rates were similar compared to after 10 days.^44^ Additionally, percutaneous tracheostomy in COVID-19 patients, including those on ECMO, is associated with earlier ventilation wean and ICU discharge.^6,45^ Our team performed tracheostomy a mean of 25.8 days from intubation, which is somewhat longer than recommendations advising assessment of tracheostomy candidacy within 10 to 14 days of intubation.^46^ This potential delay in tracheostomy insertion is likely secondary to the ill-defined prognosis and heterogeneity of this patient cohort, in addition to multiple medical comorbidities experienced by patients during the third wave of the pandemic. We performed a comparison of clinical outcomes in Kohne et al^44^ with our findings (Table 4). With adequate experience and training, a percutaneous approach is associated with reduced operative time and hospital costs, in addition to similar patient safety profile.^6,7,47^ In COVID-19 patients on ECMO, apneic percutaneous tracheostomy can assist with limiting perioperative viral aerosolization and was not associated with an adverse changes in ventilation settings.^48^ Moreover, there has been no evidence to suggest higher rates of healthcare worker COVID-19 transmission in patients who undergo percutaneous tracheostomy.^6,49^

**Table 4.** Comparison of tracheostomy outcomes in COVID-19 patients on ECMO.

Study	No. of patients	Mortality (%)	Time to ECMO decannulation	Perioperative bleeding (%)
This study	24	8 (33.3)	38.9 ± 15.2^*^	3 (12.5)
Kohne et al^44^	2259	1128 (50)	27.9 (17.3-43.0)^**^	208 (9)

Abbreviations: ECMO, extracorporeal membrane oxygenation.

* Mean ± standard deviation.

** Median (interquartile range).

The primary limitation of this study is the lack of cohort matching. We also have only included COVID-19 patients on ECMO in our analysis as our institution performed few tracheostomies in ECMO patients prior to the onset of the pandemic. These findings are also limited by the quality and accuracy of the data entered into to the electronic medical record system.

Based on this study, we suggest holding ECMO-associated anticoagulation 24 hours prior to and after tracheostomy in order to reduce the likelihood of perioperative bleeding events in patients with no additional contraindications to withholding anticoagulation. Consistent with other literature, percutaneous tracheostomy appears to be a safe option in most COVID-19 patients on ECMO, with future work potentially benefiting from high-quality prospective studies with larger patient cohorts.

## Supplemental Material

sj-docx-1-aor-10.1177_00034894231166648 – Supplemental material for Tracheostomy in Critically Ill COVID-19 Patients on Extracorporeal Membrane Oxygenation: A Single-Center Experience Click here for additional data file. Supplemental material, sj-docx-1-aor-10.1177_00034894231166648 for Tracheostomy in Critically Ill COVID-19 Patients on Extracorporeal Membrane Oxygenation: A Single-Center Experience by Phillip Staibano, Shahzaib Khattak, Faizan Amin, Paul T. Engels and Doron D. Sommer in Annals of Otology, Rhinology & Laryngology
