# Supplementary material for: Tracheostomy in Critically Ill COVID-19 Patients on Extracorporeal Membrane Oxygenation: A Single-Center Experience
Source: Ann Otol Rhinol Laryngol. 2023 Apr 9;132(12):1520–7. doi: 10.1177/00034894231166648 (PMC10086820; doi:10.1177/00034894231166648)
Supplement: sj-docx-1-aor-10.1177_00034894231166648 – Supplemental material for Tracheostomy in Critically Ill COVID-19 Patients on Extracorporeal Membrane Oxygenation: A Single-Center Experience [file sj-docx-1-aor-10.1177_00034894231166648.docx]

| **Table S1.** Summary of all included patients (N = 24) | | | | | | | | | | |  |  |  |  |  |  |  |
| --- | --- | --- | --- | --- | --- | --- | --- | --- | --- | --- | --- | --- | --- | --- | --- | --- | --- |
|  | **Demographics** | | | **COVID-19** | | **Tracheostomy details** | | | | **ECMO** | | | | | | |  |
| **Patient No.** | **Age (years)** | **Gender** | **BMI (kg/m^2^)** | **Variants of concern** | **Vaccine status** | **Type** | **Time to tracheostomy (days)** | **Time to decannulation (days)** | **Perioperative complications** | **Duration (days)** | **Time from intubation (days)** | **Mean aPTT (SD) (seconds)** | **Mean platelets (SD) (10^9^/L)** | **Mean anti-Xa (U/mL) (SD)** | **Mean INR (SD)** | **Mean fibrinogen (g/L) (SD)** | **Clinical status** |
| 1 | 45 | Male | 24.9 | None | None | PCT | 32 | 27 | None | 27 | 3 | 58.4 (21.7) | 111 (38.6) | 0.46 (0.21) | 1.20 (0.09) | 2.92 (1.26) | Alive |
| 2 | 37 | Female | 26.6 | None | None | PCT | 14 | 47 | None | 16 | 5 | 70.6 (22.3) | 176 (43) | 0.44 (0.30) | 1.26 (0.11) | 4.6 (1.05) | Alive |
| 3 | 52 | Female | 32.7 | None | None | OT | 19 | NR | None | 28 | 3 | 58.8 (44.2) | 90.5 (48.2) | 0.50 (2.13) | 1.18 (2.39) | 5.19 (2.05) | Alive |
| 4 | 58 | Male | NR | None | None | PCT | 30 | NA | None | NA | 8 | 58.1 (70.1) | 143 (77.8) | 0.55 (1.26) | 1.14 (1.33) | 2.88 (1.62) | Deceased |
| 5 | 53 | Male | 28.1 | NR | NR | OT | 13 | NA | None | NA | 4 | 37.6 (21.4) | 150 (43.2) | 0.42 (0.32) | 1.20 (0.12) | 3.40 (1.60) | Deceased |
| 6 | 43 | Male | 28.2 | S, N501Y S | None | PCT | NR | NR | None | 37 | NR | 58.7 (24.1) | 92.1 (30.2) | 0.68 (0.22) | 1.57 (0.41) | 1.94 (1.13) | Alive |
| 7 | 49 | Male | 30.4 | S, N501Y S | None | PCT | 22 | NA | None | NA | 4 | 45.1 (18.6) | 92.8 (41.3) | 0.44 (0.21) | 1.37 (0.27) | 3.47 (1.38) | Deceased |
| 8 | 53 | Male | 30.1 | None | None | PCT | 25 | 55 | None | 48 | 10 | 48.3 (15.7) | 116 (25.9) | 0.95 (0.49) | 1.18 (0.15) | 4.12 (3.29) | Alive |
| 9 | 52 | Female | 20.1 | S | None | PCT | 21 | 50 | NR | 33 | 1 | 67.7 (20.8) | 131 (36.4) | 0.49 (0.17) | 1.20 (0.07) | 3.67 (1.64) | Alive |
| 10 | 46 | Female | 54.8 | S, N501Y S, B.1.1.7 | None | OT | 65 | 58 | POD1: Severe bleed controlled with absorbable packing and electrocautery | 82 | 1 | 36.9 (17.1) | 158 (74.6) | 0.29 (0.14) | 1.21 (0.37) | 2.84 (1.04) | Alive |
| 11 | 34 | Male | 24.3 | S, N501Y S | NR | PCT | 26 | NA | POD0: Mild bleed controlled with digital pressure | NA | 4 | 37. 9 (17.9) | 104 (27.5) | 0.40 (0.13) | 1.19 (0.11) | 2.32 (1.08) | Deceased |
| 12 | 52 | Male | 27.1 | N501Y S | None | PCT | 18 | NA | None | NA | 7 | 70.6 (37.5) | 120 (53) | 0.35 (0.18) | 1.27 (0.12) | 3.41 (1.26) | Deceased |
| 13 | 38 | Male | 34.8 | B1.617.2 | None | PCT | 30 | NR | POD1: Moderate bleed controlled with absorbable packing | 46 | 9 | 61.1 (13.8) | 159 (19.3) | 0.36 (0.17) | 1.10 (0.04) | 4.74 (0.70) | Alive |
| 14 | 52 | Female | 33.5 | B1.617.2 | None | PCT | 14 | 41 | None | 36 | 3 | 55.9 (17.9) | 163 (58.6) | 0.35 (0.14) | 1.17 (0.09) | 3.20 (1.36) | Alive |
| 15 | 63 | Male | 32.3 | B1.617.2 | None | PCT | 29 | NA | None | NA | 6 | 70.3 (24.2) | 155 (42.6) | 0.37 (0.18) | 1.22 (0.11) | 2.74 (1.21) | Deceased |
| 16 | 51 | Male | NR | B1.617.2 | None | PCT | 36 | 90 | None | 38 | 12 | 64.6 (32.7) | 125 (57.7) | 0.38 (0.27) | 1.36 (0.21) | 4.47 (2.26) | Alive |
| 17 | 41 | Male | NR | B1.617.2 | None | PCT | 17 | 30 | None | 21 | 3 | 40.4 (9.44) | 209 (103) | 0.34 (0.20) | 1.05 (0.09) | 3.09 (0.90) | Alive |
| 18 | 48 | Male | NR | B1.617.2 | None | PCT | 26 | 62 | none | 47 | 6 | 47.5 (13.6) | 147 (47.2) | 0.31 (0.13) | 1.1 (0.08) | 3.47 (0.91) | Alive |
| 19 | 47 | Male | NR | B1.617.2 | None | PCT | 21 | 57 | None | 45 | 3 | 39.4 (11.7) | 113 (36.6) | 0.38 (0.16) | 1.11 (0.07) | 3.14 (0.67) | Alive |
| 20 | 59 | Male | 32.7 | B1.617.2 | None | PCT | 22 | NA | None | NA | 4 | 63 (16) | 147 (30.5) | 0.35 (0.13) | 1.33 (0.26) | 3.45 (1.89) | Deceased |
| 21 | 45 | Female | 54.7 | B1.617.2 | None | OT | 28 | 24 | None | 29 | 7 | 38.1 (10.1) | 139 (41.9) | 0.23 (0.11) | 1.08 (0.08) | 2.83 (0.88) | Alive |
| 22 | 58 | Male | 31.1 | B1.617.2 | None | PCT | 14 | 83 | None | 50 | 7 | 55 (16.1) | 272 (82.5) | 0.35 (0.19) | 1.04 (0.07) | 5.49 (1.79) | Alive |
| 23 | 54 | Male | 32.4 | B1.617.2 | None | PCT | 38 | 18 | None | 40 | 16 | 45.7 (22.2) | 127 (57.9) | 0.41 (0.19) | 1.33 (0.14) | 2.50 (1.15) | Alive |
| 24 | 56 | Male | 29.5 | B1.617.2 | None | PCT | 34 | 27 | None | 27 | 4 | 35.2 (9.40) | 150 (32.3) | 0.27 (0.17) | 1.23 (0.15) | 2.77 (1.67) | Deceased |
| BMI, body mass index; VOC, variants of concern; NA, Not applicable; NR, not reported; OT, open tracheostomy; PCT, percutaneous tracheostomy; POD, postoperative day | | | | | | | | | | |  |  |  |  |  |  |  |
